# Identification of linear B-cell epitopes on the gE envelope protein of varicella-zoster virus

**DOI:** 10.1128/spectrum.00723-25

**Published:** 2025-09-02

**Authors:** Yan Niu, Jingming Zhou, Yankai Liu, Haili Wang, Hongliang Liu, Yumei Chen, Xifang Zhu, Chao Liang, Aiping Wang

**Affiliations:** 1 School of Life Sciences, Zhengzhou University 12636, Zhengzhou, Henan, China; 2 Longhu Laboratory 693032 , Zhengzhou, Henan, China; Oklahoma State University College of Veterinary Medicine, Stillwater, Oklahoma, USA

**Keywords:** varicella-zoster virus, gE envelope protein, B-cell epitopes

## Abstract

Varicella-zoster virus (VZV) is a highly infectious DNA virus with an envelope. It is the pathogen causing varicella and herpes zoster. The glycoprotein E (gE) envelope protein, a primary target for VZV diagnostic reagents and vaccines, is highly immunogenic and plays a critical role in the pathogenicity of VZV. However, the B-cell epitopes and immunodominant regions of gE remain poorly characterized. In this study, we utilized anti-VZV gE monoclonal antibodies to localize antigenic sequences of gE at ^101^VYNQGRGIDSGERLMQPTQM^120^, ^124^EDLGDDTGIHVI^135^, and ^141^DDRHKIVNVDQRQYGDVFKGD^161^ by the overlapping peptide method. Results from conservative and structural analysis revealed that ^101^VYNQGRGIDSGERLMQPTQM^120^ was a highly conserved epitope and exposed on the surface of VZV gE. These findings provide important information for researching the VZV diagnostic reagents and may facilitate further efforts to design VZV vaccines.

**IMPORTANCE:**

VZV infection can cause varicella (commonly in children) and herpes zoster (in adults). Currently, the prevention of VZV primarily relies on vaccination. The currently approved vaccines to prevent varicella are live-attenuated viruses that carry the risk of inducing herpes zoster. Therefore, there is an urgent need to develop safer novel VZV vaccines. The gE protein, the most abundant and highly conserved glycoprotein on the VZV envelope, exhibits strong immunogenicity and can induce neutralizing antibodies, making it a critical target for developing novel VZV vaccines and diagnostic reagents. To date, research on the B-cell epitopes of the VZV gE protein remains limited, particularly regarding the identification of conformational B-cell epitopes. This project aims to identify linear B-cell epitopes of the gE protein, providing a scientific foundation for the development of novel VZV vaccines based on epitopes.

## INTRODUCTION

Varicella-zoster virus (VZV), a highly infectious and human-specific pathogen, is the causative agent of varicella (chickenpox) and herpes zoster (HZ) (shingles) (1). Primary VZV infection causes varicella, which is generally a mild, self-limiting disease in children with the highest incidence rate in the 5–9 age group (2). However, in immunocompromised patients and susceptible individuals, particularly pregnant women, varicella can be more serious and even life-threatening (3–5). Following the resolution of varicella, VZV establishes lifelong latency in sensory ganglia (6). When immunity wanes in adults, reactivation of VZV can lead to HZ with severe pain and post-herpetic neuralgia (PHN) (7).

VZV exhibits a recognized serotype, with 99.8% sequence conservation across five identified phylogenetic clades (1, 8). A report described a second wild-type VZV serotype which had lost a major B-cell epitope defined on the glycoprotein E (gE) ectodomain and had been recovered in the United States, Canada, and Sweden (9–11). The envelope of VZV contains at least nine glycoproteins, which are highly immunogenic and closely related to the pathogenicity of the virus. Among them, gE, encoded by ORF68, is the most abundant envelope protein and the major immunoprotective antigen, with key roles in possibly host-cell entry, viral replication, virion envelopment, and cell-to-cell virus transmission (12–14). The gE antibody could neutralize VZV with the presence of a complement (15). Therefore, gE is the main research target for VZV vaccine and detection reagent. To date, the three-dimensional structure of the VZV gE protein has been resolved (16). VZV gE could form heterodimers with gI. Inhibition of gE/gI heterodimerization could result in decreased gE maturation and surface expression, inhibiting gI incorporation into virions and thus blocking infection of skin and T cells *in vivo* (1).

Several vaccines have been approved for varicella prevention, reducing incidence rates by over 80% (17), associated hospitalizations by 88% (18), and varicella deaths by 66% (19). Furthermore, the use of a live attenuated HZ vaccine has markedly reduced 61.1% of the illness burden, 66.5% of PHN incidence rates, and 51.3% of HZ incidence rates in adults over 60 years old (20). Additionally, a gE subunit vaccine combined with the AS01B adjuvant system has been approved for herpes zoster prevention and may reduce 97.2% of HZ incidence rates in adults over 50 years old (12).

In this study, we focused on the VZV gE protein as the research target and verified its B-cell epitopes by immunological experiments. The identification of B-cell epitopes of the VZV gE protein lays a foundation for the development of VZV diagnostic reagents and novel epitope-focused vaccines.

## MATERIALS AND METHODS

### Cells, plasmids, and reagents

DH5α, BL21, and Rosetta competent cells were purchased from GenScript (Nanjing, China). The pET-28a and pET-32a plasmids were stored in our laboratory. The gE ectodomain and anti-VZV gE monoclonal antibodies (mAbs) were prepared in our laboratory (21). All chemical reagents used in the experiments were obtained from commercial sources and of analytical grade.

### Identification of anti-VZV gE mAbs

The EC_50_ of mAbs was measured by indirect enzyme-linked immunosorbent assay (ELISA). Ninety-six-well ELISA plates were coated with 200 ng of gE diluted in carbonate-bicarbonate buffer (pH 9.6, 0.05 M) and incubated at 37℃ for 2 h. Then, the plates were washed with phosphate-buffered saline with Tween 20 (PBST) three times and saturated with 200 µL of 5% skim milk at 4℃ overnight. After washing three times, the plates were incubated with serial twofold dilutions of mAbs (100 µL/well) at 37℃ for 30 min. After washing five times, the wells were added with horseradish peroxidase (HRP)-conjugated goat antimouse antibody (100 µL/well) and incubated at 37℃ for 30 min. After washing five times, the reactions were developed using tetramethylbenzidine for 5 min and stopped with 2 M sulfuric acid. The absorbance was measured immediately at 450 nm and the EC_50_ was calculated by GraphPad.

The subtypes of mAbs were measured by a commercial mouse mAb subtype identification kit.

### Construction and expression of pET-28a-P1, P2, P3, P4, P5, and P6

The optimized VZV gE ectodomain gene (GenBank No. MF898328.1) was synthesized by Sangon Biotech Co., Ltd. Six pairs of primers were designed to amplify the P1–P6 genes. Then, the genes were cloned into the pET-28a vector to construct the recombinant plasmids pET-28a-P1–P6, respectively. Furthermore, the recombinant plasmids were transformed into DH5α competent cells and confirmed to be constructed correctly by gene sequencing analysis. Finally, the pET-28a-P1–P6 recombinant plasmids were transformed into BL21 competent cells and expressed by isopropyl β-D-1-thiogalactopyranoside induction.

### Identification of truncated proteins P1–P6

The reactions between the truncated proteins P1–P6 and mAbs were identified by Western blot. The Western blot was as follows. The recombinant proteins P1–P6 and the gE protein were resolved by 12% SDS-PAGE gels and transferred to polyvinylidene fluoride (PVDF) membrane. The PVDF membrane was saturated with 5% skimmed milk at 4°C overnight. Then, the membrane was incubated with the anti-VZV gE mAb (1:1,000 dilution in phosphate-buffered saline) at 37°C for 1 h. After washing with PBST three times, the membrane was incubated with HRP-conjugated goat antimouse IgG (H + L) (1:5,000 dilution in 5% skimmed milk) at 37°C for 1 h. After washing three times, the blots were developed by enhanced chemiluminescence.

The reactions between the truncated proteins P1–P6 and mAbs were identified by dot blot and indirect ELISA, respectively. The dot blot was the same as the Western blot except that the recombinant proteins P1–P6 and the gE protein were placed on the nitrocellulose membrane. The indirect ELISA was the same as the ELISA steps above, except that the recombinant proteins P1–P6 and the gE protein were coated on the ELISA plate.

### Expression and identification of pET-32a-P2-1-5

The VZV gE-P2 protein was divided into five segments, named P2-1-5, each overlapping 10 amino acids. Five pairs of primers were designed to amplify the P2-1-5 gene. Then the genes were cloned into the pET-32a vector to construct the recombinant plasmid pET-32a-P2-1-5, respectively. Furthermore, the recombinant plasmids were transformed into DH5α competent cells for amplification and then transformed into Rosetta competent cells for expression. Similarly, the reactions between the truncated protein P2-1-5 and mAbs were identified by Western blot, dot blot, and indirect ELISA, respectively.

### Synthesis and identification of peptides

Nine peptides divided from gE-P2-2, gE-P2-3, and gE-P2-4 were synthesized. Then the B-cell epitopes of the gE protein were identified by dot blot and indirect ELISA, respectively.

### Prediction and analysis of the tertiary structure of the VZV gE protein

AlphaFold3 is one of the most advanced tools developed for protein structure prediction. It can still predict protein structure accurately and regularly in the absence of homologous structure (22). In this study, AlphaFold3 was used to predict the tertiary structure of the VZV gE protein. Then, the B-cell epitopes of the VZV gE protein were displayed on the tertiary structure.

## RESULTS

### Identification of anti-VZV gE mAbs

**Fig 1:**
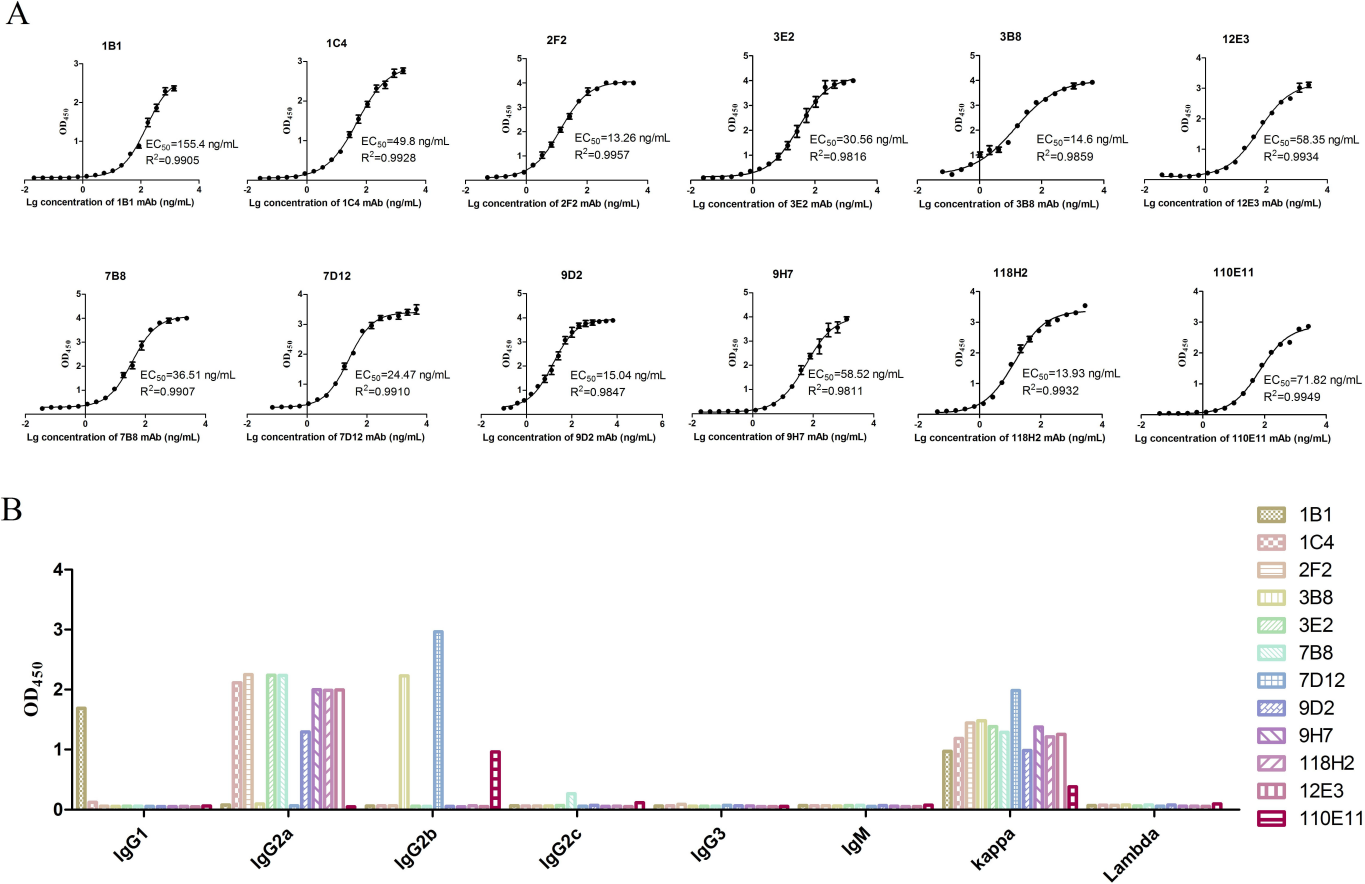
Characterizations of anti-VZV gE mAbs. (**A**) The EC_50_ of mAbs was measured by indirect ELISA. (**B**) The subtype of mAbs was measured by a commercial mouse mAb subtype identification kit.

The EC_50_ values of the mAbs were determined using indirect ELISA and analyzed with GraphPad software. As shown in Fig. 1A, the EC_50_ of 2F2 mAb was lowest (13.26 ng/mL), indicating that the 2F2 mAb had a higher affinity with the VZV gE protein. The heavy chain subtypes of mAbs (Fig. 1B) mainly were IgG1, IgG2a, and IgG2b. All of the light chain subtypes were kappa.

### Truncated expression and identification of the VZV gE protein

**Fig 2:**
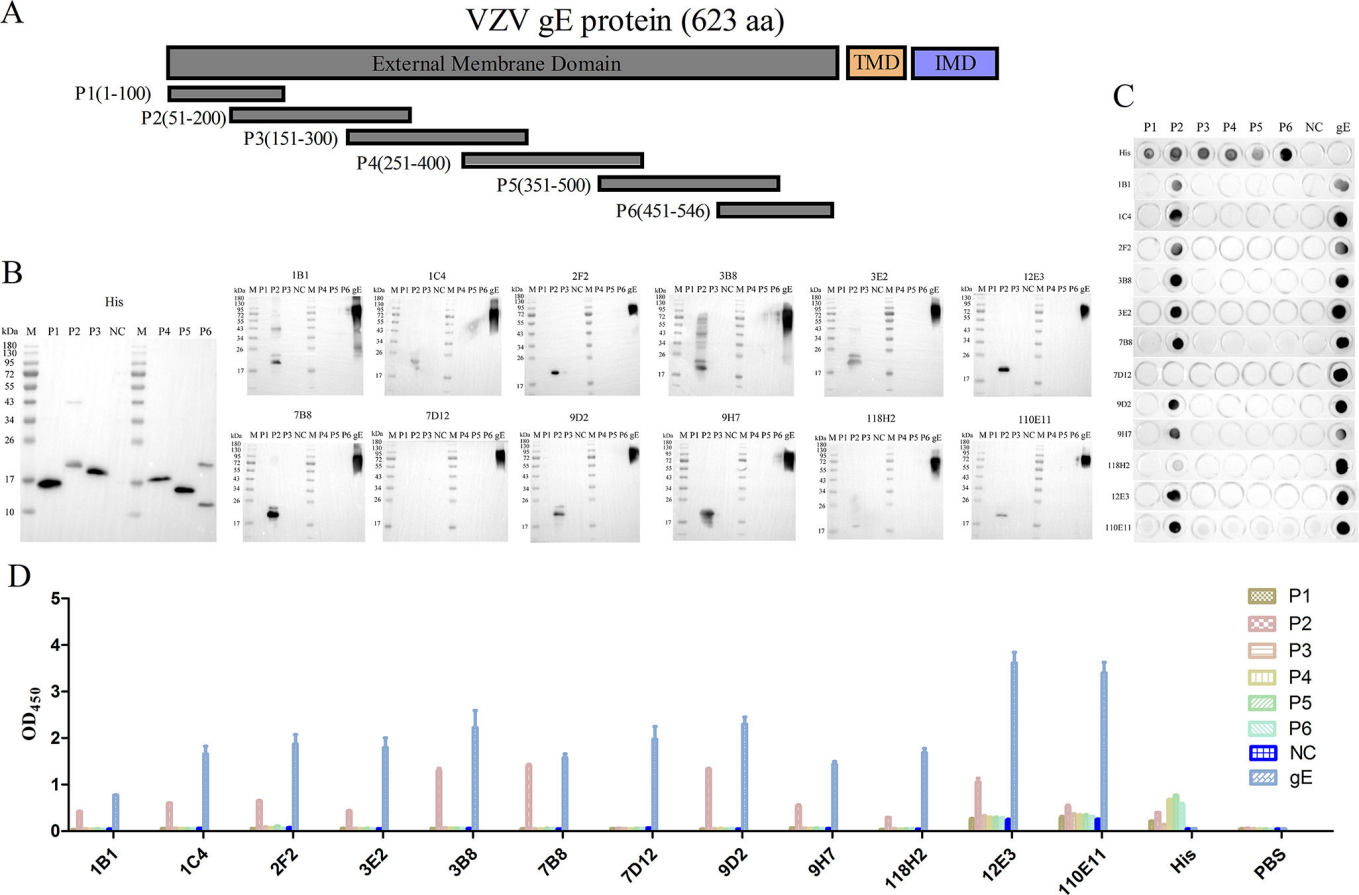
Truncated expression and identification of VZV gE ectodomain. (**A**) Truncated diagram of gE ectodomain. The reactions between the truncated proteins P1–P6 and mAbs were identified by (B) Western blot, (**C**) dot blot, and (**D**) indirect ELISA.

The VZV gE ectodomain was divided into six segments, named P1–P6, each overlapping 50 amino acids (Fig. 2A). After prokaryotic expression of recombinant proteins P1–P6 with His tag, expression supernatants were collected for further identification of B-cell epitopes. The results of Western blot (Fig. 2B), dot blot (Fig. 2C), and indirect ELISA (Fig. 2D) showed that recombinant proteins P1–P6 were recognized by His mAb. Notably, the recombinant protein P2 exhibited reactivity with multiple mAbs, including 1B1, 1C4, 2F2, 3B8, 3E2, 12E3, 7B8, 9D2, 9H7, and 110E11, indicating that the P2 protein had high immune reactivity. In addition, no recombinant protein could be recognized by 7D12 mAb, which showed that the 7D12 might recognize a conformational epitope. Moreover, the 118H2 mAb had a weak reaction with P2 protein and needed to be further confirmed.

### Truncated expression and identification of the P2 protein

**Fig 3:**
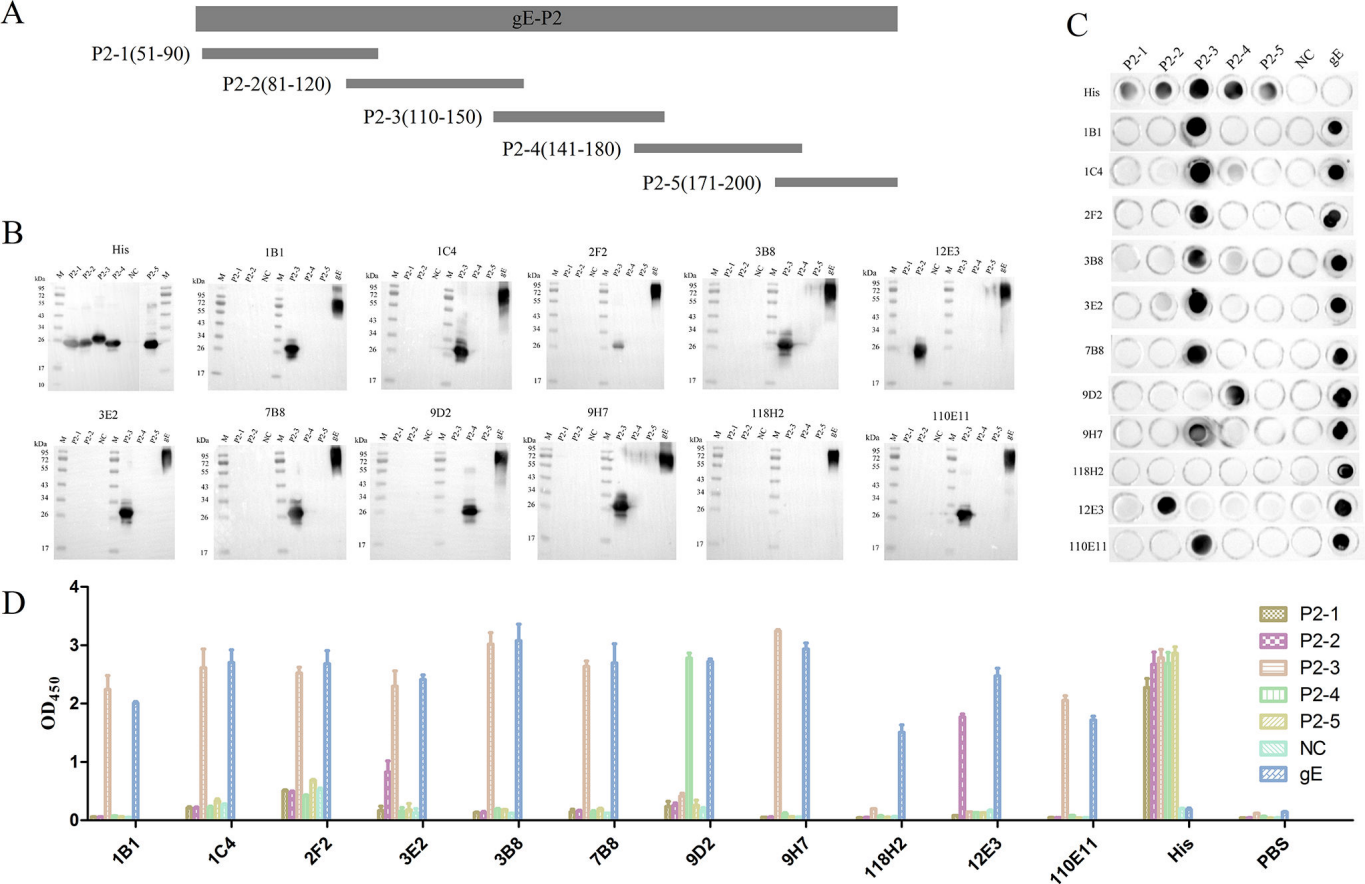
Truncated expression and identification of the P2 protein. (**A**) Truncated diagram of P2 protein. The reactions between the truncated proteins P2-1-5 and mAbs were identified by (B) Western blot, (**C**) dot blot, and (**D**) indirect ELISA.

Furthermore, the P2 protein was divided into five segments, named P2-1-5, each overlapping 10 amino acids (Fig. 3A), and the truncated proteins with His tag were obtained by prokaryotic expression. Similarly, the reaction between the truncated protein P2-1-5 and mAbs was identified by Western blot (Fig. 3B), dot blot (Fig. 3C), and indirect ELISA (Fig. 3D). The identification results showed that the truncated protein P2-1-5 was recognized by His mAb. Interestingly, no recombinant protein could be recognized by 118H2 mAb, which showed that the 118H2 mAb might recognize a conformational epitope. In addition, the truncated protein P2-3 could be recognized by 1B1, 1C4, 2F2, 3B8, 3E2, 7B8, 9H7, and 110E11 mAb, indicating that the 110−150 aa of the gE protein was the immunodominant region. Moreover, the recombinant protein P2-2 could be recognized by 12E3 mAb, and P2-4 could be recognized by 9D2 mAb.

### Synthesis and identification of peptides

**Fig 4:**
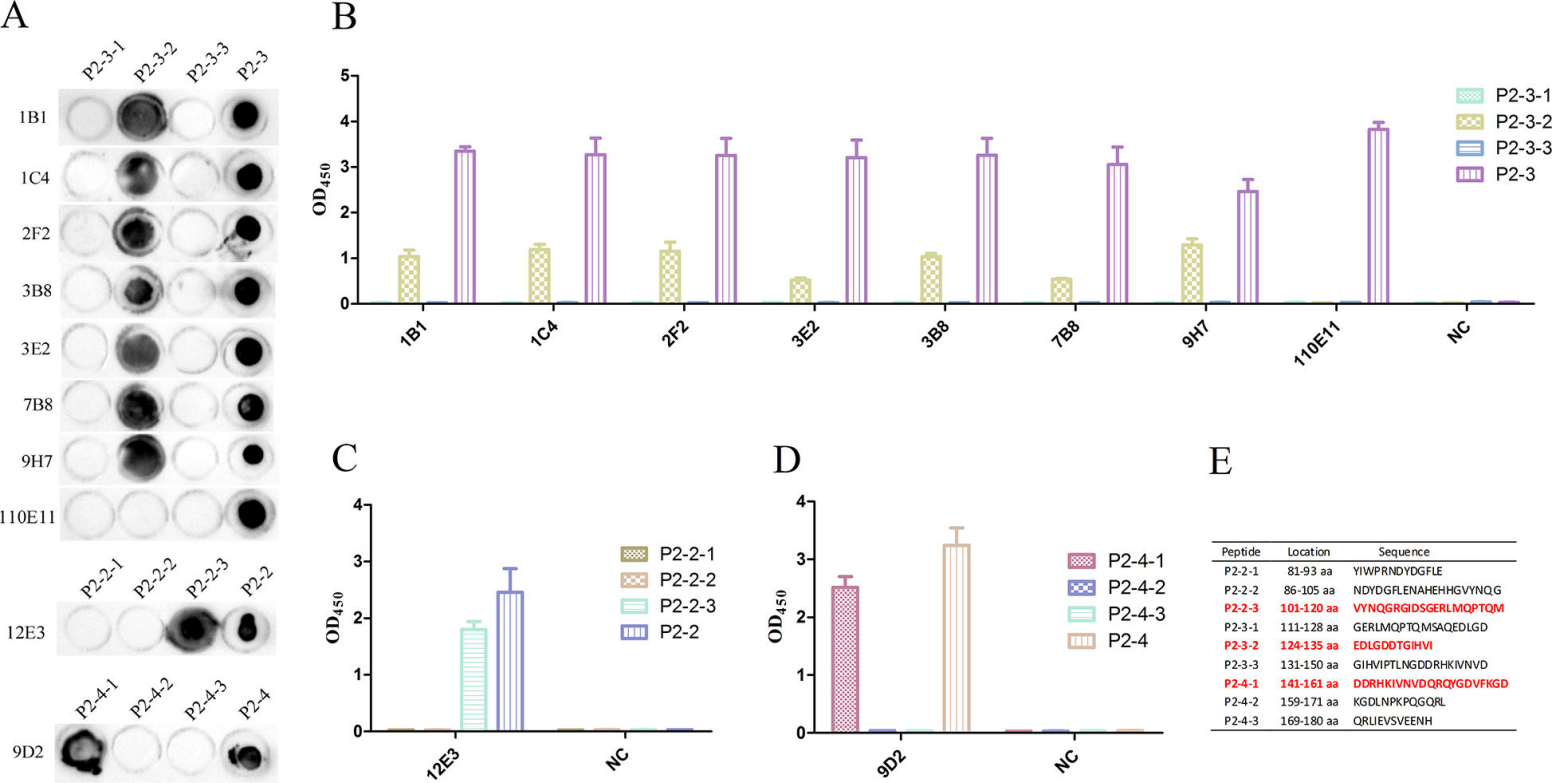
Synthesis and identification of peptides. (**A**) The reactions between the synthetic peptides and mAbs were identified by dot blot. (**B**) The reactions between the synthetic peptides P2-3-1-3 and mAbs were identified by indirect ELISA. (**C**) The reactions between the synthetic peptides P2-2-1-3 and 12E3 mAb were identified by indirect ELISA. (**D**) The reactions between the synthetic peptides P2-4-1-3 and 9D2 mAb were identified by indirect ELISA. (**E**) Synthetic peptide sequence.

Nine peptides divided from gE-P2-2, gE-P2-3, and gE-P2-4 were synthesized as shown in Fig. 4E. The reactions between the synthetic peptides and mAbs were identified by dot blot and indirect ELISA, respectively. As shown in Fig. 4A and B, the peptide P2-3-2 could be recognized by 1B1, 1C4, 2F2, 3B8, 3E2, 7B8, and 9H7 mAb. Interestingly, no peptide could be recognized by 110E11 mAb, which showed that the 110E11 mAb might recognize a conformational epitope. In addition, both the results of dot blot (Fig. 4A) and indirect ELISA (Fig. 4C) showed that the peptide P2-2-3 could be recognized by 12E3 mAb. Moreover, the peptide P2-4-1 could be recognized by 9D2 mAb, identified by dot blot (Fig. 4A) and indirect ELISA (Fig. 4D). Finally, as shown in Fig. 4E, the B-cell epitopes of the VZV gE protein were located on antigenic sequences ^101^VYNQGRGIDSGERLMQPTQM^120^, ^124^EDLGDDTGIHVI^135^, and ^141^DDRHKIVNVDQRQYGDVFKGD^161^.

### Prediction of the VZV gE protein structure

**Fig 5:**
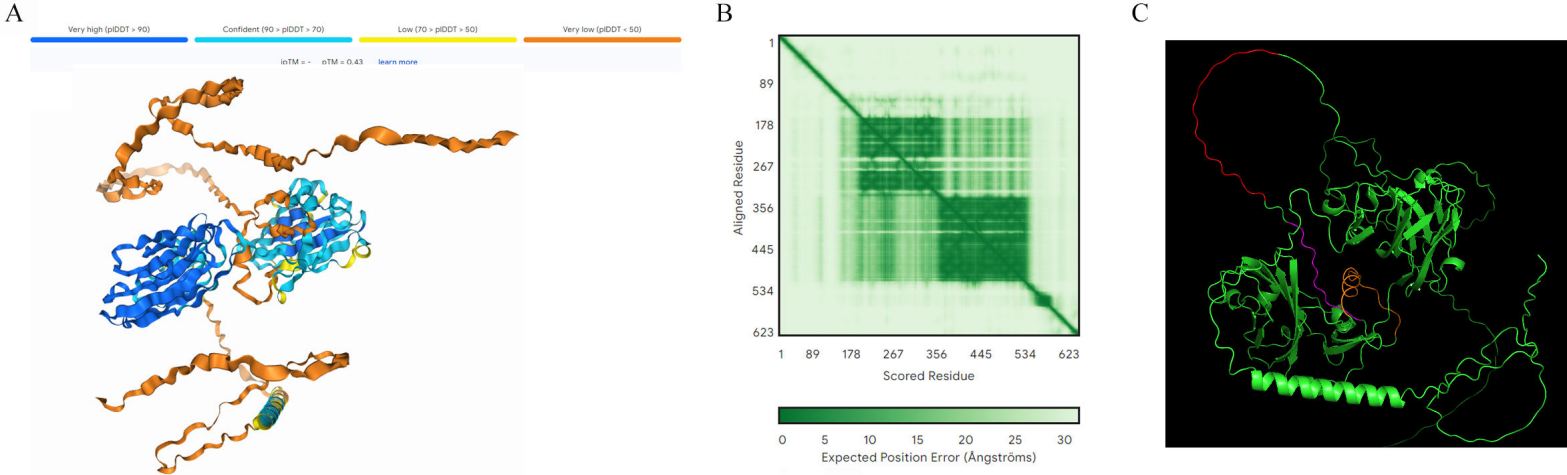
Tertiary structure prediction results of the gE protein. (**A**) Tertiary structure of the gE protein (all 623 amino acids). (**B**) Confidence analysis. (**C**) The location of the polypeptide.

The tertiary structure of the VZV gE protein was analyzed by AlphaFold3, and the result was shown in Fig. 5A, where the cool color represented high confidence and the red represented low confidence or “possible disorder prediction.” The prediction result showed that most amino acid residues at positions 1–170 and 570–623 at the N terminal of the VZV gE protein were in a random coiled state with loose structure and located on the protein surface. The remaining amino acid residues were mostly in α-helix and β-folded states, and their morphology was relatively fixed, mainly in the protein interior. The result of confidence analysis (Fig. 5B) showed that the prediction reliability of 170–570 amino acid residues at the N terminal of the gE protein was relatively high. The confidence at both ends was low and may be in a disordered state. As shown in Fig. 5C, the epitope ^101^VYNQGRGIDSGERLMQPTQM^120^ was dyed in red; the epitope ^124^EDLGDDTGIHVI^135^ was dyed in purple; and the epitope ^141^DDRHKIVNVDQRQYGDVFKGD^161^ was dyed in orange. Among them, ^101^VYNQGRGIDSGERLMQPTQM^120^ was a highly conserved epitope and exposed on the surface of VZV gE.

## DISCUSSION

VZV infection can cause varicella and herpes zoster, causing great disease burden (23). The development of a safe and efficient vaccine is one of the effective measures to prevent VZV infection. The epitope-focused vaccines have become a hot spot and trend in vaccine development due to their strong pertinency and slight side effects (24). A study proposes a polyvalent multiepitope subunit vaccine targeting six glycoproteins that are crucial for VZV infection (25). The identification methods of antigen epitopes mainly include X-ray diffraction technique, protein digestion, immunological experiment, and bioinformatics prediction. In this study, we selected the frequently used immunological experiment to study the antigen epitopes of the VZV gE protein.

The epitope 141–161 was already described (9). Another paper demonstrates a new phenotype for the virus with a mutation in the gE epitope. Moreover, these mutated viruses have also been found in Canada and Sweden (10, 11, 26). Previous studies have shown that amino acids 1–134 and 101–161 of gE were the most antigenic, and other fragments (spanning residues 161–623) had weak or no antigenicity. Pepscan analysis revealed three immunodominant sequences (residues 56–75, 86–105, and 116–135) detected using sera from both varicella and zoster patients. All sera from varicella patients reacted strongly with an epitope in peptides 66–85 but not zoster. A complement-dependent neutralizing epitope was identified in residues 71–90 (27). Subsequent research showed that the dominant B-cell epitopes were found in peptides spanning amino acids 41–60, 56–75, 101–120, 116–135, 131–150, and 141–161 (28). Another study revealed two complement-dependent neutralizing epitopes in residues 121–135 (29) and 341–355 (30). In this study, using anti-VZV gE mAbs, we identified antigenic sequences of gE at ^101^VYNQGRGIDSGERLMQPTQM^120^, ^124^EDLGDDTGIHVI^135^, and ^141^DDRHKIVNVDQRQYGDVFKGD^161^ by the overlapping peptide method. Results from conservative and structural analysis showed that ^101^VYNQGRGIDSGERLMQPTQM^120^ was a highly conserved epitope and exposed on the surface of VZV gE. These findings offer valuable insights for the development of VZV diagnostic reagents and may facilitate further efforts to design VZV vaccines.

## References

[1] Zerboni L, Sen N, Oliver SL, Arvin AM. 2014. Molecular mechanisms of varicella zoster virus pathogenesis. Nat Rev Microbiol 12:197–210. doi:10.1038/nrmicro3215 24509782 PMC4066823

[2] Luan G, Yao H, Yin D, Liu J. 2024. Trends and age-period-cohort effect on incidence of varicella under age 35 - China, 2005-2021. China CDC Wkly 6:390–395. doi:10.46234/ccdcw2024.076 38737482 PMC11082652

[3] Charlier C, Le Mercier D, Salomon LJ, Ville Y, Kermorvant-Duchemin E, Frange P, Postaire M, Lortholary O, Lecuit M, Leruez-Ville M. 2014. Varicella-zoster virus and pregnancy. Presse Med 43:665–675. doi:10.1016/j.lpm.2014.04.001 24863663

[4] Harger JH, Ernest JM, Thurnau GR, Moawad A, Momirova V, Landon MB, Paul R, Miodovnik M, Dombrowski M, Sibai B, Van Dorsten P. 2002. Risk factors and outcome of varicella-zoster virus pneumonia in pregnant women. J Infect Dis 185:422–427. doi:10.1086/338832 11865393

[5] Tunbridge AJ, Breuer J, Jeffery KJM. 2008. Chickenpox in adults - clinical management. J Infect 57:95–102. doi:10.1016/j.jinf.2008.03.004 18555533

[6] Zhu H, Zheng C, Xing J, Wang S, Li S, Lin R, Mossman KL. 2011. Varicella-zoster virus immediate-early protein ORF61 abrogates the IRF3-mediated innate immune response through degradation of activated IRF3. J Virol 85:11079–11089. doi:10.1128/JVI.05098-11 21835786 PMC3194975

[7] Ilyas S, Chandrasekar PH. 2020. Preventing varicella-zoster: advances with the recombinant zoster vaccine. Open Forum Infect Dis 7:ofaa274. doi:10.1093/ofid/ofaa274 32760747 PMC7392035

[8] Breuer J. 2010. VZV molecular epidemiology. Curr Top Microbiol Immunol 342:15–42. doi:10.1007/82_2010_9 20229231

[9] Santos RA, Padilla JA, Hatfield C, Grose C. 1998. Antigenic variation of varicella zoster virus Fc receptor gE: loss of a major B cell epitope in the ectodomain. Virology (Auckl) 249:21–31. doi:10.1006/viro.1998.9313 9740773

[10] Tipples GA, Stephens GM, Sherlock C, Bowler M, Hoy B, Cook D, Grose C. 2002. New variant of varicella-zoster virus. Emerg Infect Dis 8:1504–1505. doi:10.3201/eid0812.020118 12498673 PMC2738511

[11] Wirgart BZ, Estrada V, Jackson W, Linde A, Grose C. 2006. A novel varicella-zoster virus gE mutation discovered in two Swedish isolates. J Clin Virol 37:134–136. doi:10.1016/j.jcv.2006.06.007 16905355

[12] Dendouga N, Fochesato M, Lockman L, Mossman S, Giannini SL. 2012. Cell-mediated immune responses to a varicella-zoster virus glycoprotein E vaccine using both a TLR agonist and QS21 in mice. Vaccine (Auckl) 30:3126–3135. doi:10.1016/j.vaccine.2012.01.088 22326899

[13] Gershon AA, Gershon MD. 2010. Perspectives on vaccines against varicella-zoster virus infections. Curr Top Microbiol Immunol 342:359–372. doi:10.1007/82_2010_12 20232192 PMC5391036

[14] Berarducci B, Ikoma M, Stamatis S, Sommer M, Grose C, Arvin AM. 2006. Essential functions of the unique N-terminal region of the varicella-zoster virus glycoprotein E ectodomain in viral replication and in the pathogenesis of skin infection. J Virol 80:9481–9496. doi:10.1128/JVI.00533-06 16973553 PMC1617235

[15] Sullivan NL, Reuter-Monslow MA, Sei J, Durr E, Davis CW, Chang C, McCausland M, Wieland A, Krah D, Rouphael N, Mehta AK, Mulligan MJ, Pulendran B, Ahmed R, Vora KA. 2018. Breadth and functionality of varicella-zoster virus glycoprotein-specific antibodies identified after zostavax vaccination in humans. J Virol 92:e00269-18. doi:10.1128/JVI.00269-18 29743372 PMC6026762

[16] Harshbarger WD, Holzapfel G, Seraj N, Tian S, Chesterman C, Fu Z, Pan Y, Harelson C, Peng D, Huang Y, Chandramouli S, Malito E, Bottomley MJ, Williams J. 2024. Structures of the Varicella Zoster virus glycoprotein E and epitope mapping of vaccine-elicited antibodies. Vaccines (Basel) 12:1111. doi:10.3390/vaccines12101111 39460278 PMC11511291

[17] Seward JF. 2002. Varicella disease after introduction of varicella vaccine in the United States, 1995-2000. JAMA 287:606. doi:10.1001/jama.287.5.606 11829699

[18] Zhou F. 2005. Impact of Varicella vaccination on health care utilization. JAMA 294:797. doi:10.1001/jama.294.7.797 16106004

[19] Nguyen HQ, Jumaan AO, Seward JF. 2005. Decline in mortality due to varicella after implementation of varicella vaccination in the United States. N Engl J Med 352:450–458. doi:10.1056/NEJMoa042271 15689583

[20] Oxman MN, Levin MJ, Johnson GR, Schmader KE, Straus SE, Gelb LD, Arbeit RD, Simberkoff MS, Gershon AA, Davis LE. 2005. A vaccine to prevent herpes zoster and postherpetic neuralgia in older adults. N Engl J Med 352:2271–2284. doi:10.1056/NEJMoa051016 15930418

[21] Wang A, Niu Y, Zhao J, Liu H, Ding P, Chen Y, Zhou J, Zhu X, Zhang Y, Liang C, Zhang G. 2023. Rapid detection of varicella-zoster virus based on an immunochromatographic strip. Virology (Auckl) 586:35–42. doi:10.1016/j.virol.2023.07.008 37481958

[22] Abramson J, Adler J, Dunger J, Evans R, Green T, Pritzel A, Ronneberger O, Willmore L, Ballard AJ, Bambrick J. 2024. Accurate structure prediction of biomolecular interactions with AlphaFold 3. Nature New Biol 630:493–500. doi:10.1038/s41586-024-07487-w PMC11168924 38718835

[23] Zheng Q, Wang D, Lin R, Chen Y, Huang H, Xu Z, Zheng C, Xu W. 2023. Mendelian randomization analysis suggests no associations of human herpes viruses with amyotrophic lateral sclerosis. Front Neurosci 17:1299122. doi:10.3389/fnins.2023.1299122 38156274 PMC10754516

[24] Correia BE, Bates JT, Loomis RJ, Baneyx G, Carrico C, Jardine JG, Rupert P, Correnti C, Kalyuzhniy O, Vittal V. 2014. Proof of principle for epitope-focused vaccine design. Nature New Biol 507:201–206. doi:10.1038/nature12966 PMC4260937 24499818

[25] Amin Rani N, Moin AT, Patil R, Barketullah Robin T, Zubair T, Nawal N, Sami MRS, Morshed MM, Zhai J, Xue M, Hossain M, Zheng C, Abul Manchur M, Islam NN. 2023. Designing a polyvalent vaccine targeting multiple strains of varicella zoster virus using integrated bioinformatics approaches. Front Microbiol 14:1291868. doi:10.3389/fmicb.2023.1291868 38075876 PMC10704101

[26] Santos RA, Hatfield CC, Cole NL, Padilla JA, Moffat JF, Arvin AM, Ruyechan WT, Hay J, Grose C. 2000. Varicella-zoster virus gE escape mutant VZV-MSP exhibits an accelerated cell-to-cell spread phenotype in both infected cell cultures and SCID-hu mice. Virology (Auckl) 275:306–317. doi:10.1006/viro.2000.0507 10998331

[27] Fowler WJ, Garcia-valcarcel M, Hill-perkins MS, Murphy G, Harper DR, Jeffries DJ, Burns NR, Adams SE, Kingsman AJ, Layton GT. 1995. Identification of immunodominant regions and linear B cell epitopes of the gE envelope protein of varicella-zoster virus. Virology (Auckl) 214:531–540. doi:10.1006/viro.1995.0064 8553555

[28] Garcia-Valcarcel M, Fowler WJ, Harper DR, Jeffries DJ, Layton GT. 1997. Induction of neutralizing antibody and T-cell responses to Varicella-zoster virus (VZV) using Ty-virus-like particles carrying fragments of glycoprotein E (gE). Vaccine (Auckl) 15:709–719. doi:10.1016/S0264-410X(96)00228-9 9178473

[29] Zhu R, Liu J, Chen C, Ye X, Xu L, Wang W, Zhao Q, Zhu H, Cheng T, Xia N. 2016. A highly conserved epitope-vaccine candidate against varicella-zoster virus induces neutralizing antibodies in mice. Vaccine (Auckl) 34:1589–1596. doi:10.1016/j.vaccine.2016.02.007 26873057

[30] Liu J, Zhu R, Ye X, Yang L, Wang Y, Huang Y, Wu J, Wang W, Ye J, Li Y, Zhao Q, Zhu H, Cheng T, Xia N. 2015. A monoclonal antibody-based VZV glycoprotein E quantitative assay and its application on antigen quantitation in VZV vaccine. Appl Microbiol Biotechnol 99:4845–4853. doi:10.1007/s00253-015-6602-5 25935343

